# Utilization of a new technology of 3D biliary CT for ERCP-related procedures: a case report

**DOI:** 10.1186/s12876-020-01304-0

**Published:** 2020-05-24

**Authors:** Masao Toki, Hidekatsu Tateishi, Tsubasa Yoshida, Koichi Gondo, Shunsuke Watanabe, Tadakazu Hisamatsu

**Affiliations:** 1 Department of Gastroenterology and Hepatology, Kyorin University School of Medicine, Shinkawa 6-20-2, Mitaka-shi, Tokyo, 181-8611 Japan; 2 Department of Radiology, Kyorin University School of Medicine, Shinkawa 6-20-2, Mitaka-shi, Tokyo, 181-8611 Japan

**Keywords:** Endoscopic retrograde cholangiopancreatography (ERCP), Malignant hilar biliary obstruction, Three-dimensional (3D) images

## Abstract

**Background:**

Endoscopic retrograde cholangiopancreatography (ERCP) is still performed using two-dimensional (2D) X-ray images. The success rate and risk of complications are considered operator-dependent. We explored performing an ERCP-related procedure with 3D-computed tomography (CT) biliary imaging for preoperative simulation and intraoperative reference in a patient with malignant biliary obstruction.

**Case presentation:**

The patient was a 66-year-old man who underwent rectal resection and postoperative chemotherapy for rectal cancer. A liver metastasis caused obstructive jaundice and acute cholangitis, necessitating emergency hospitalization. A 3.5 cm mass in the hilar region of the biliary tree caused type IV biliary obstruction according to the Bismuth-Corlette classification of hilar cholangiocarcinoma. ERCP and biliary drainage were performed repeatedly, but had no effect. Given that selective bile duct drainage had proven extremely difficult with the conventional procedures, three-dimensional (3D) images were created from preoperative CT image data using a 3D image reconstruction system (SYNAPSE VINCENT version 5, FUJIFILM Corporation, Tokyo, Japan). Using the 3D images for preoperative planning and intraoperative reference, biliary drainage and stent placement were successfully performed without complications. Postoperatively, the patient had no further cholangitis or need for stent replacement up to his death.

**Conclusions:**

We report the first case of an ERCP-related procedure with 3D biliary imaging for preoperative simulation and intraoperative reference in a patient with malignant biliary obstruction. The 3D image reconstruction is useful for preoperative planning and could contribute to an increased success rate, decreased complications, a shorter operation time, and reduced radiation exposure to the operator.

## Background

Biliary drainage with endoscopic retrograde cholangiopancreatography (ERCP) is generally performed using endoscopy and two-dimensional (2D) fluoroscopic image guidance. Selective bile duct drainage is often difficult in patients with malignant biliary hilar obstruction because of the complicated positional relationships of the bile ducts and bile duct obstruction patterns. The technical difficulties result in longer procedure times, resulting in increased radiation exposure to patients and endoscopists, and it may be associated with a risk of complications [1–3]. This calls for innovations for simpler and safer ERCP-related procedures.

Recent introduction of multi-detector computed tomography (MDCT) and high-speed magnetic resonance imaging (MRI) have dramatically improved visualization for formulating diagnostic and therapeutic strategies in hepatopancreaticobiliary disease. In surgery and interventional radiology, a three-dimensional (3D) image analysis system is used to construct a 3D image that is applied in preoperative planning [4, 5]. Reports have indicated that it has contributed to shortening the procedure time, improving the procedure success rate, and reducing the incidence of complications. Respiratory-triggered three-dimensional MR cholangiopancreatography (3D-MRCP) provides high-spatial-resolution images of the biliary tract and pancreatic duct, and enables MR virtual endoscopy using volume rendering to visualize the lumens of the gallbladder, bile duct, and pancreatic duct [6]. However, the utility of 3D-CT image in ERCP-related procedures has not been reported. Here, we report the first case in which we successfully performed biliary drainage using 3D-CT images for reference.

## Case presentation

**Fig. 1:**
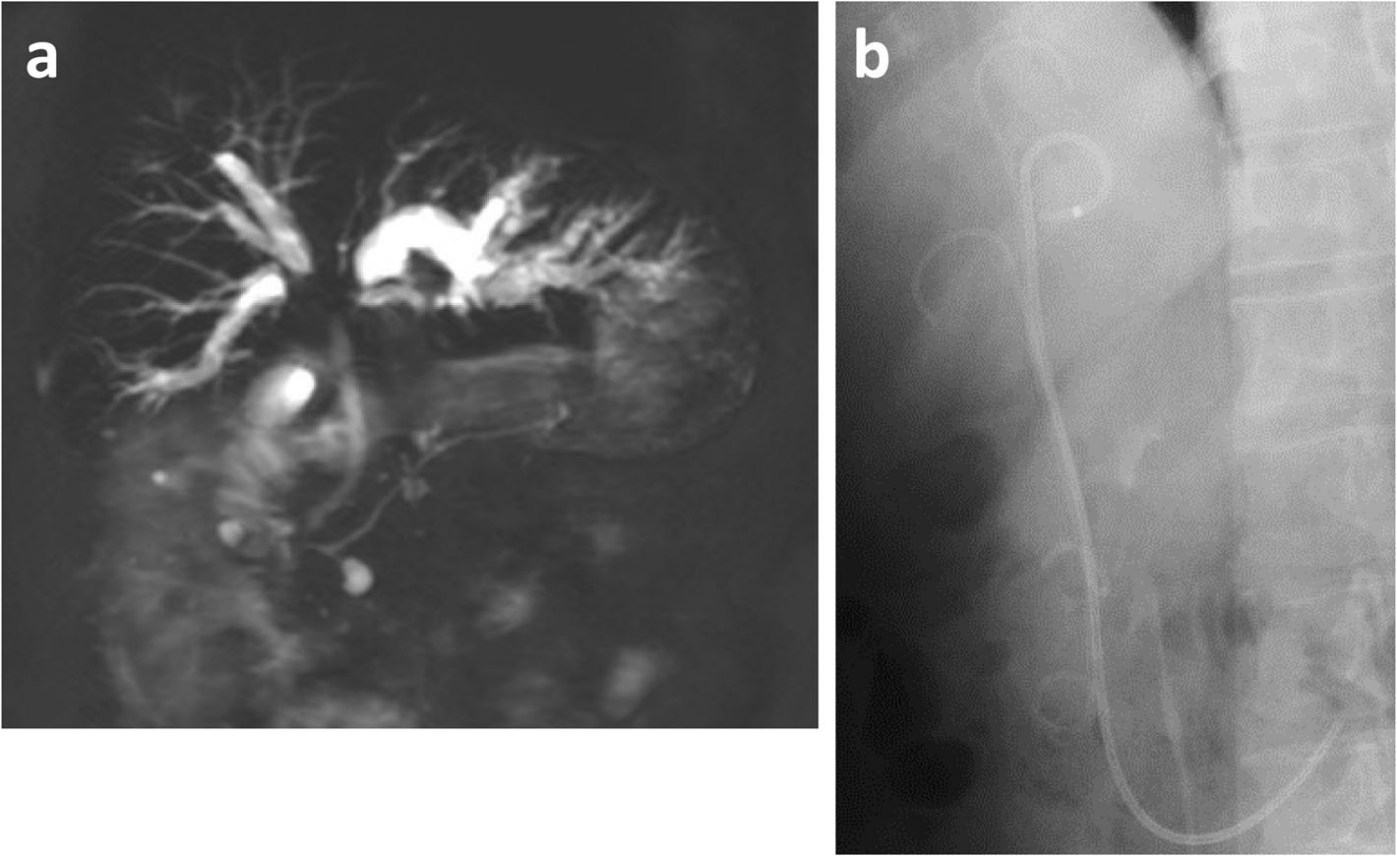
**a** Magnetic resonance imaging. A metastasis in the biliary hilar region of the liver caused type IV biliary obstruction and bilateral intrahepatic bile duct dilation. **b**: ERCP imaging. PS had already been placed in the left intrahepatic bile duct (branch of B3 and B2) and right intrahepatic bile duct (B5 and B7). ERCP, endoscopic retrograde cholangiopancreatography; PS, plastic stent

**Fig. 2:**
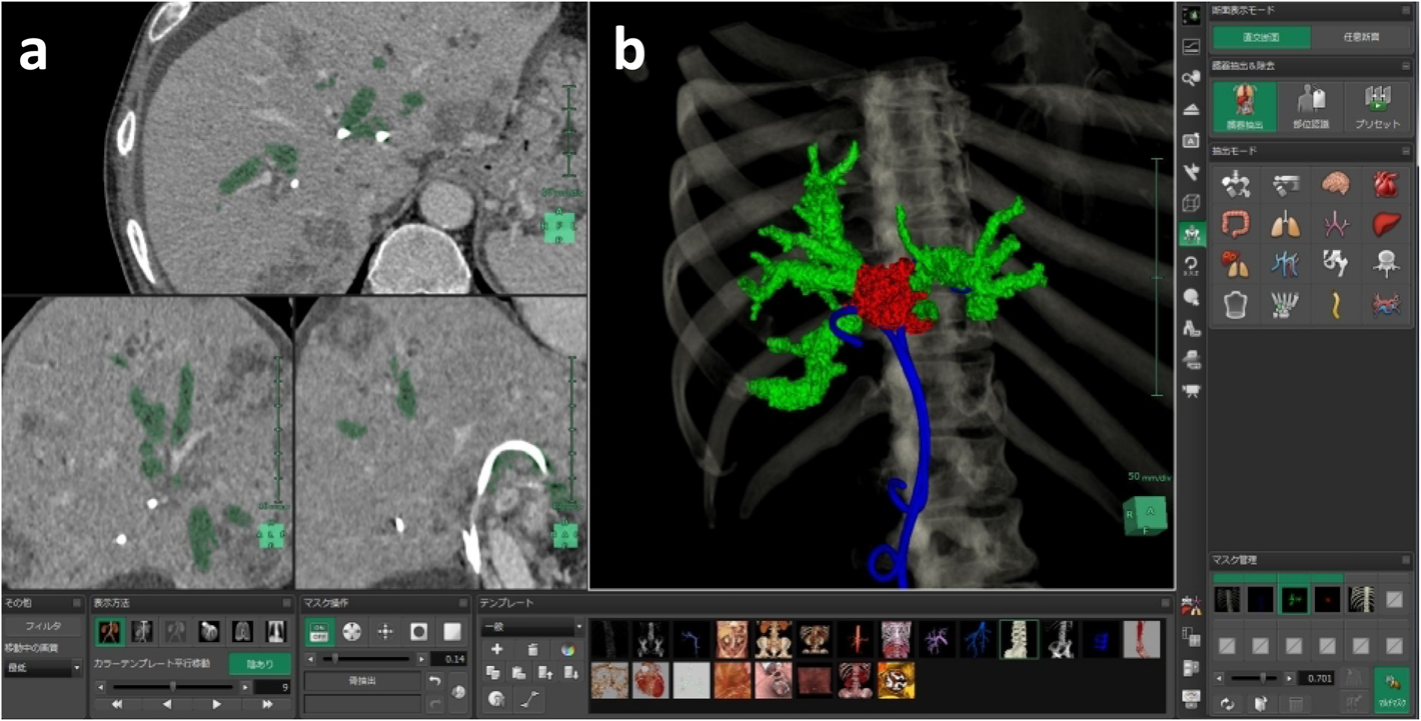
Creation of 3D images of the biliary tree from CT image data using a 3D image analysis system (SYNAPSE VINCENT version 5, FUJIFILM Corporation, Tokyo, Japan). **a**: CT images. **b**. A 3D image created by tracing dilated intrahepatic bile ducts. Green: dilated bile ducts. Red: metastatic liver tumor at hilar biliary region. Blue: PS tubes placed previously. CT, computed tomography; PS, plastic stent; 3D, three-dimensional

The patient was a 66-year-old man who underwent rectal resection and postoperative chemotherapy for rectal cancer. Bile duct obstruction due to a liver metastasis caused obstructive jaundice and acute cholangitis, which resulted in emergency hospitalization. On magnetic resonance imaging (Fig. 1a), a 3.5 cm mass in the biliary hilum caused type IV biliary obstruction according to the Bismuth-Corlette classification [7] of hilar cholangiocarcinoma. Despite several attempts at biliary drainage (branch of B3 and B2, B5, B7) via ERCP, obstructive jaundice was not improved. The patient was emergently hospitalized again because of acute cholangitis with a 39 °C fever and epigastric pain, although bile duct plastic stents (PS) had been placed in the left intrahepatic bile duct (branch of B3 and B2) and right intrahepatic bile duct (B5 and B7) (Fig. 1b). Repeated ERCP and biliary drainage were performed, had no effect as shown on the CT (Fig. 2). In this case, many of biliary branches were divided by the obstruction at hilar biliary. Only by 2D image, it was hard to identify the relation between dilated biliary branches and drainage tube placed. Given that selective bile duct drainage had proven extremely difficult with the conventional procedures, 3D images were created from preoperative CT image data using a 3D image reconstruction system (SYNAPSE VINCENT version 5, FUJIFILM Corporation, Tokyo, Japan). We used the 3D images for preoperative planning and performed biliary drainage using them as an intraoperative reference.

**Fig. 3:**
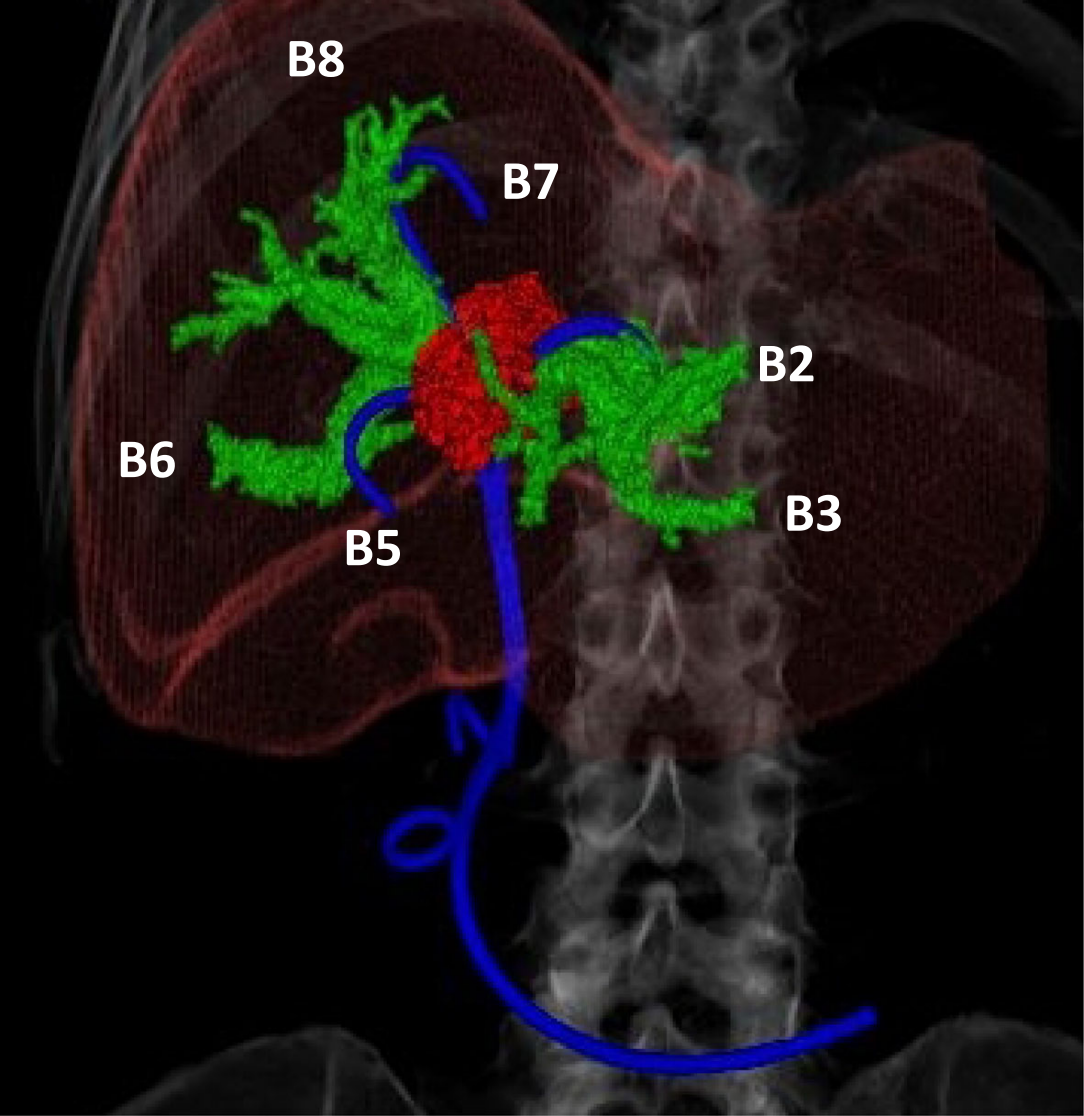
Positional relationship between the completed 3D biliary image and plastic stents. Green: dilated bile ducts. Red: metastatic liver tumor at hilar biliary region. Blue: PSs placed previously. PS, plastic stent; 3D, three-dimensional

**Fig. 4:**
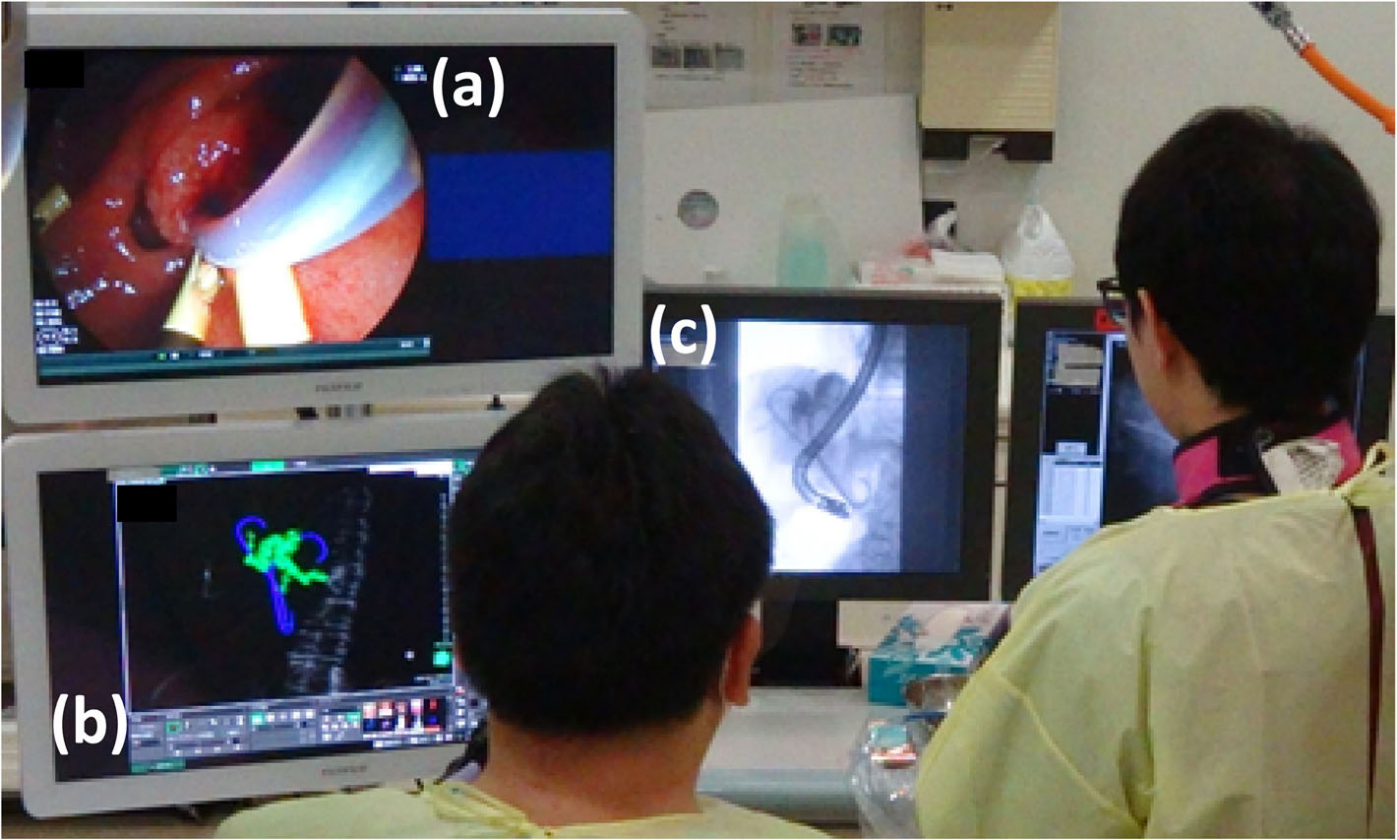
Actual ERCP procedure. Endoscopists could see the endoscope screen (**a**), a 3D image of the biliary tree, which could be rotated freely (**b**), and a 2D real-time fluoroscopic image (**c**) at the same time. ERCP, endoscopic retrograde cholangiopancreatography; 3D, three-dimensional; 2D, two-dimensional

**Fig. 5:**
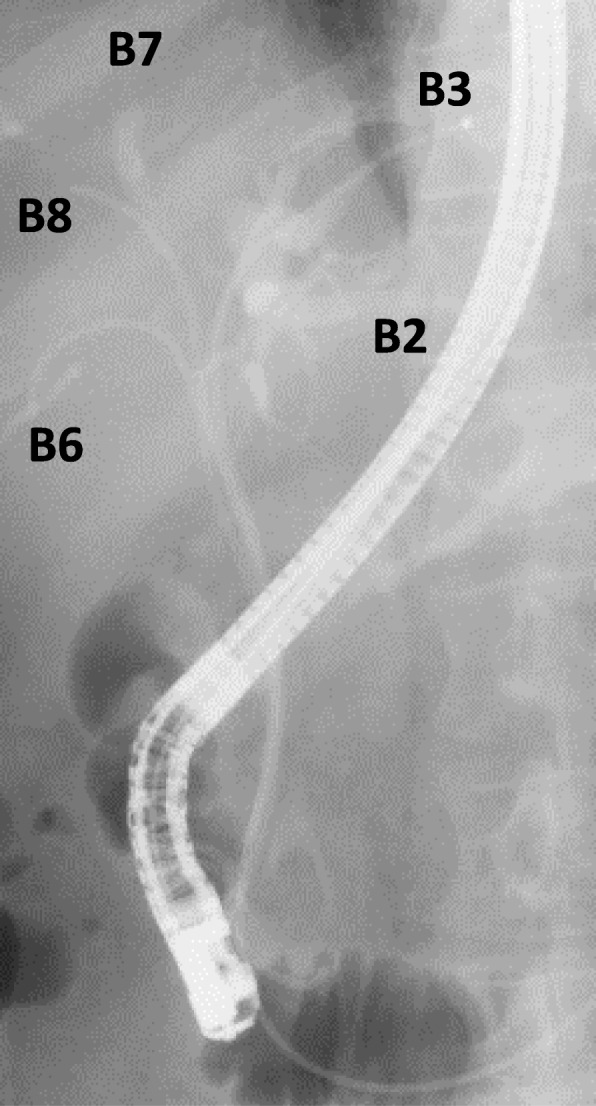
A 2D X-ray image of replaced PSs. PS, plastic stent; 2D, two-dimensional

Residual dilation was observed in the left intrahepatic bile duct (B3). Although the PS was allowing slight decompression of the left intrahepatic bile duct (B2), marked biliary dilation persisted; thus, the PS was deemed ineffective for B3, while no biliary dilation was found around the PSs placed in B5 and B7, so these were deemed to be effective. Marked dilation of B6 and B8 was noted, and drainage was deemed necessary (Fig. 3). On the basis of the above findings, we planned preoperatively that an additional PS would be placed in the deep part of B3 and several other PSs would be replaced (B6 and B8). The entire ERCP procedure was performed under the combination of fluoroscopic images of a standard side-view duodenoscope (EDT-580, FUJIFILM, Tokyo, Japan) and the 3D images. This procedure was performed using a multipurpose imaging system incorporating a C-arm (VersiFlex Apla, HITACHI corporation Tokyo, Japan). During the actual ERCP procedure, endoscopists could see the endoscope video (a), the 3D image of the bile ducts that rotate freely (b), and the 2D fluoroscopic image (c) at the same time (Fig. 4). With the 3D images for preoperative planning and intraoperative reference, biliary drainage was successfully performed without complications (Fig. 5). Postoperatively, the patient had no further cholangitis or need for stent replacement up to his death.

## Discussion and conclusions

ERCP is still performed using 2D fluoroscopy, with operator- and case-dependent success rates and complication rates. Despite the advances in fluoroscopic equipment such as a multipurpose imaging system incorporating C-arm, ERCP using 2D fluoroscopy images has room for improvement in terms of procedure times and radiation exposure for patients and medical staff.

There were cases that could be sufficiently confirmed and considered useful for preoperative planning by MRCP [8, 9]. On the other hand, 3D images could selectively depict the necessary branches of the bile duct, and in addition, the lumbar spine and tumor could be selectively described in the background, making it very easy to understand the three-dimensional positional relationship.

In this patient with malignant hilar biliary stenosis, selective bile duct drainage was technically difficult. We created the 3D images for pre-operative evaluation. The 3D images successfully identified the biliary branches that required drainage, and we were able to confirm the positional relationships of the biliary branches. We could check the created 3D image by moving it freely in any direction. The 3D images were available in the operating room, and were used for intraoperative reference during the procedure. As a result, the drainage of the target bile duct branch was successfully completed with reduced procedure time.

Demands for minimally invasive procedures for medical purposes are rapidly increasing. In the field of digestive endoscopy, various endoscopic procedures under X-ray fluoroscopic guidance are being adopted, including ERCP. The radiation exposure involved in those procedures has led to major concerns over radiation exposure [3]. ERCP poses a potential risk of radiation exposure to medical staff and patients [10, 11]. Efforts are needed to further reduce radiation exposure to patients and medical staff as recommended by the European Society of Gastrointestinal Endoscopy 2012 guidelines. The American Society for Gastrointestinal Endoscopy recommended the frequency with which fluoroscopy time and radiation dose are measured and documented as quality indicators for ERCP [10, 12].

Pre- and intra-operative 3D images can delineate the bile duct anatomy more easily and shorten the procedure time, consequently reducing radiation dose, making for a safer procedure. This tool is useful to understand localization and navigate the bile duct drainage, rather than to evaluate dilated branches precisely.

We reported here a case with bile duct obstruction due to metastatic rectal cancer successfully treated with the combination of 3D images and fluoroscopically-assisted ERCP. This approach could revolutionize existing ERCP-related procedures.

## Data Availability

The datasets used and analyzed during the current study are available from the corresponding author on reasonable request.

## Abbreviations

ERCP: Endoscopic retrograde cholangiopancreatography
3D: Three-dimensional
2D: Two-dimensional
CT: Computed tomography
MRI: Magnetic resonance imaging
MDCT: Multi-detector computed tomography
PS: Plastic stent
